# Normal values for the Rhinoplasty Outcome Evaluation (ROE) questionnaire

**DOI:** 10.1590/S1808-86942012000400015

**Published:** 2015-10-20

**Authors:** Suemy Cioffi Izu, Eduardo Macoto Kosugi, Karen Vitols Brandão, Alessandra Stanquini Lopes, Leonardo Bomediano Sousa Garcia, Vinícius Magalhães Suguri, Luis Carlos Gregório

**Affiliations:** 1 MD. Otorhinolaryngologist - Paulista School of Medicine - Federal University of São Paulo (UNIFESP-EPM); Fellowship in Rhinology - UNIFESP-EPM; 2 MSc in Sciences; Preceptor - Head of the Otorhinolaryngology Residency Program UNIFESP-EPM; 3 MD - UNIFESP-EPM; 3rd-Year ENT Resident - UNIFESP-EPM; 4 MSc in Sciences - UNIFESP-EPM; Assistant Physician - Department of Rhinology; 5 PhD in Medicine - UNIFESP-EPM; Vice-Chair - Department of Otorhinolaryngology and Head and Neck Surgery - UNIFESP-EPM. Department of Rhinology - Rhinolaryngology Course - Department of ENT-HNS -UNIFESP-EPM

**Keywords:** quality of life, questionnaires, reference values, rhinoplasty

## Abstract

Rhinoplasty Outcome Evaluation (ROE) is an easy-to-use questionnaire that allows comprehensive assessment of rhinoplasty-related patient satisfaction. However, normal values for this questionnaire are not known.

**Objective**: To translate and cross-culture adapt the Rhinoplasty Outcome Evaluation questionnaire to Brazilian Portuguese and to establish normality parameters.

**Materials and Methods**: Cross-sectional study with ROE administration to 62 patients waiting for rhinoplasty or septorhinoplasty (Cases) and 100 volunteer subjects without desire or need for nasal surgery (Controls). Assessment of possible sensitivity and specificity cutoffs.

**Results**: The cases' mean score was 6.6 or 27.5% (SD 3.18; min 0; max 15) and controls' mean score was 17.94 or 74.75% (SD 3.91; min 7; max 24). The best cutoff was 12 or 50%, with 95.16% sensitivity and 95% specificity.

**Conclusion**: At the zero-to-24 score of the Brazilian Portuguese ROE, we found 12 as the best cutoff, with 95.16% of sensitivity and 95% of specificity.

## INTRODUCTION

Result assessment in rhinoplasty is not usually systematized in descriptions of series - both Brazilian and international. Great emphasis is placed on the surgical technique, approach, complications, sequelae and rates of second-look procedures; however, very little has been studied about the assessment of the final rhinoplasty result, especially from the patient's viewpoint^1^. One very interesting way to estimate surgical results from the viewpoint of patient satisfaction is by means of quality-of-life questionnaires^2^. Especially in rhinoplasty - a procedure which aims at impacting directly on patient satisfaction with his/her own image and, consequently, self-esteem. The validity of such questionnaires is unquestionable.

Quality-of-life can be defined as the individual's perception of his situation in life, within the cultural and value system in which he lives, and in relation to one's objectives, expectations, standards and concerns^3^. In other words, it is indeed a very encompassing concept, of a holistic image of fullness. Aesthetic interventions, when assessed under such glasses, are means aiming at reaching such state of wholeness. Thus, an attractive way to estimate rhinoplasty results would be to investigate how much quality of life such procedure was able to give those submitted to it.

Based on such philosophy, Alsarraf^4^ created a series of questionnaires in order to specifically assess the results from facial aesthetic procedures from the view point of patient satisfaction. In creating the questions, the author took into account the main factors with influence patient satisfaction concerning the surgery: the physical issue -investigated by patient satisfaction concerning nasal shape and function; the emotional issue - estimated by the degree of confidence and desire to change appearances; and the social factor, assessed by social, professional and family acceptance. Considering that most aesthetic procedures are not carried out in an academic setting, one highly considered point when creating the questions is the ease and comfort in employing them, which enables its use also in private practice^3^. The series of questionnaires produced by Alsarraf includes four modes, each one being specific for a facial surgical procedure. The ROE (*Rhinoplasty Outcomes Evaluation)* was created in order to estimate rhinoplasty results, it is made up of six questions, two for each factor considered key in patient satisfaction (physical, emotional and social)^4^.

To impose normality criteria in aesthetics is no simple task. The need for surgery is not based on objective criteria, but rather in the subjective assessment of surgeon and patient, a determining factor to indicate or not a surgical procedure. Questionnaires can be used in order to better quantify this subjective assessment. Therefore, we need to establish the clinical relevance for the data produced by the questionnaire, in other words, the questionnaire's result must be clinically translated into “sick or not sick”, or even, “normal or altered”. This clinical relevance may be established by determining a cutting point - a questionnaire's normality parameter, so that we may classify the result into normal or altered. For instance, with a normality parameter we may help the surgeon in the decision whether or not to operate the patient, and it may also serve in the post-op to help quantify the patient's improvement.

The goal of this study is to translate and culturally adapt the *Rhinoplasty Outcome Evaluation* questionnaire into Brazilian Portuguese, and establish a normality parameter.

## METHOD

The *Rhinoplasty Outcome Evaluation* (ROE) was translated and adapted according to criteria from Guillemin et al.^5^. The ROE questionnaire has six questions, each one with five answer options, graded from zero to four. Therefore, the questionnaire score may vary between zero and 24. In order to make understanding easier, the score obtained must be divided by 24 and multiplied by 100, which leads to a score varying between zero and 100, and the higher the score, the greater is the patient's satisfaction with the nose surgery.

The questionnaires were administered by two of the authors in two different groups: Rhinoplasty Group, made up of patients with indication of rhinoplasty or rhinoseptoplasty awaiting surgery; and Control Group, made up of healthy volunteers who did not want to or did not have an indication for rhinoplasty or rhinoseptoplasty. All the subjects were volunteers and signed the Informed Consent Form, according to the protocol approved by the Ethics in Research Committee of our Institution, under Protocol number CEP 1791/11.

The scores obtained by the administration of the questionnaires were submitted to a descriptive analysis and analysis of the distribution curve, in order to check the normality. Afterwards, we checked the distribution of the scores between the groups by the ***t*** test. Then we made the response distribution graphs in a way to identify possible cutting scores to establish normal values. Using these possible scores, we calculated the questionnaire's sensitivity and specificity to obtain the ideal cutting point.

The distribution of the ages between the groups was also assessed by the ***t*** test, while the distribution of genders between the groups was assessed by the ξ^2^ test.

## RESULTS

The translation of the ROE questionnaire from English into Portuguese, according to the Guillemin criteria, resulted in the form presented in Chart 1.

**Chart 1:** ROE Questionnaire (Portuguese-BR).

Question 1: Do you like how your nose looks?
Absolutely no (0), A little (1), More or less (2), Very much (3), Absolutely yes (4)
Question 2: Do you breathe well through your nose?
Absolutely no (0), A little (1), More or less (2), Very much (3), Absolutely yes (4)
Question 3: Do you believe your friends and people who are dear to you like your nose?
Absolutely no (0), A little (1), More or less (2), Very much (3), Absolutely yes (4)
Question 4: Do you think the current appearance of your nose hampers your social or professional activities?
Always (0), Frequently (1), Sometimes (2), Rarely (3), Never (4)
Question 5: Do you think your nose looks as good as it could be?
Absolutely no (0), A little (1), More or less (2), Very much (3), Absolutely yes (4)
Question 6: Would you undergo surgery to change the appearance of your nose or to improve your breathing?
Certainly yes (0), Very likely yes (1), Possibly yes (2), Probably no (3), Certainly no (4)

We administered the questionnaires to 100 individuals happy with their noses and 62 patients waiting for a rhinoplasty or rhinoseptoplasty. The characteristics of the subjects in the study and the scores obtained are presented on Table 1. There were no statistically significant differences in the distribution of ages and genders between the groups.

**Table 1:** Descriptive analysis.

Data	Cases	Controls
Subjects		
Number	62	100
Mean age in years	32.39	30.79
Females n(%)	28 (45.16)	56 (56)
Points		
Mean	6.6	17.94
Standard deviation	3.18	3.91
Minimum	0	7
Median	6	18
Maximum	15	24
Statistical analysis:		
Ages	Test *t*	*p* = 0.40
Genders	Test ξ^2^	*p* = 0.18
Points	Test *t*	*p* < 0.0001

**Graph 1:**
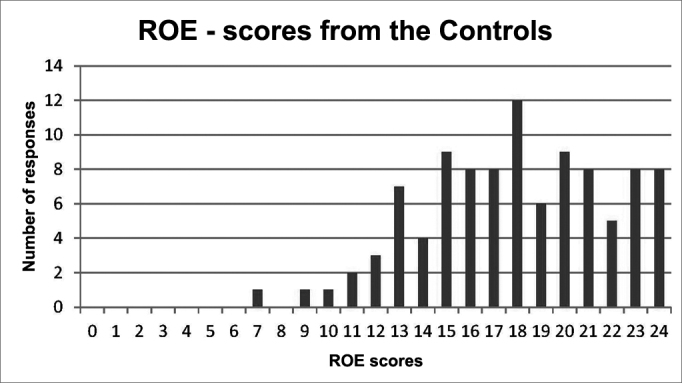
Distribution of the Control Group scores.

**Graph 2:**
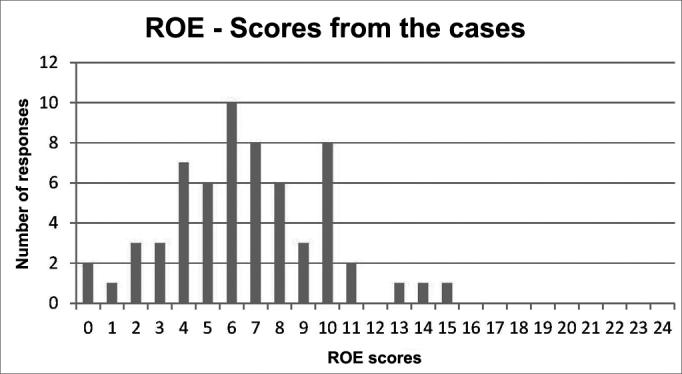
Distribution of the Rhinoplasty Group scores.

**Graph 3:**
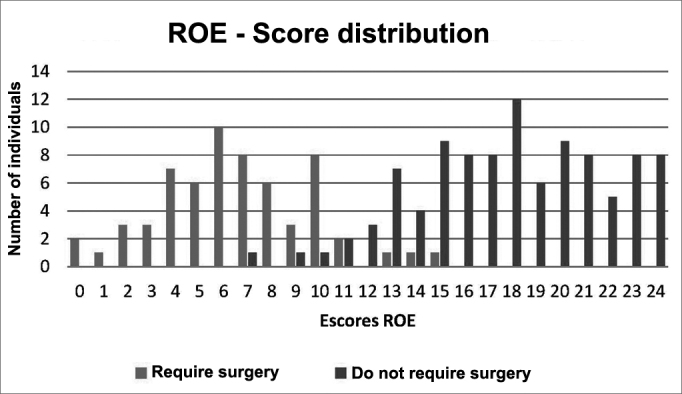
Intersection of the Control and Rhinoplasty Groups Distribution.

The score distribution in the two groups had a curve tending to normality, with sample asymmetry and kurtosis between -2 and +2. However, the groups had presented significant differences as to the values (*p* < 0.0001). The distribution of subjects according to the scores can be seen in Graph 1, Graph 2. The intersection of the distributions of the two groups can be seen on Graph 3.

Looking at Graph 3, we can see that the intersection of the curves is closer to score 11. We calculated the questionnaire's sensitivity and specificity considering the values of 10 and 14 as cutting points for normality, shown on Table 2.

**Table 2:** Variation in sensitivity and specificity.

	Maximum normality limit				
Characteristic	10	11	12	13	14
Sensitivity	79.03%	91.94%	95.16%	95.16%	96.77%
Specificity	98%	97%	95%	92%	85%

Considering Table 2, the value of 12 points is the one which presented the best ratio between sensitivity and specificity. Therefore, it is the value to be considered as the minimum limit for normality. Dividing it by 24 and multiplying it by 100, we obtain 50%. This means that values of 50% or more in the ROE questionnaire may be considered as normal or happy subjects. Values below 50% may be considered as abnormal and unhappy subjects.

## DISCUSSION

The easy administration of the questionnaire was one of the concerns Alssaraf had in making it^4^. We noticed that, after its translation, this characteristic was not lost. Part of the questionnaires can be self-administered, and just a few minutes are enough to fill out the questions - not causing discomfort for the responder. Nonetheless, patients tend to prefer the questionnaire to be administered as an interview^6^. This method is usually fast in filling out and has a lower rate of missing data, being also preferred in Brazilian validation studies^2^.

Analyzing our results, we notice two very distinct groups. The mean value of the scores between patients requiring surgery was 6.6, while the mean value of the Control Group was 17.94. There was also very little value intersection between the groups, which facilitated the identification of a cutting point, with good sensitivity and specificity indices.

The improvement brought about by surgery can be estimated by the score differences before and after surgery, as well as by the changing or not changing the classification based on the cutting point. When we utilized our minimum normality value and categorized the patients from a study by Faidiga et al.^7^, who used the ROE to assess rhinoplasty results in the long run in an academic setting, we noticed that of the 62 patients submitted to surgery, only six remained in the group of unhappy patients^7^. Employing our ROE normality criteria in another study, carried out by Arima et al.^1^, also in an academic setting, we notice that 18 of the 19 patients (94.7%) in rhinoplasty pre-op would fit as altered ROE values, while only two patients (10.5%) in the post-op would continue with altered values^1^. This shows that the cutting score established in our study seems to truly fit the questionnaire.

Considering that rhinoplasty is the aesthetic surgery that has the lowest satisfaction rate^8^, identifying good candidates to the procedure - task which is usually difficult for the surgeon, is fundamental to obtain good results. Although not being necessary for indicating surgery, the classification of patients as being candidates or not to the procedure, by using a normality value, may predict results which are more or less satisfactory. Patients with high scores in the pre-op may not be very pleased after the surgery, and they may even have a risk of worsening in their initial situation^1^.

Although some studies, besides the present one, used translated versions into Portuguese (BR)^1^, ^7^, ROE has not yet being validated to our language. The large difference in values between our Rhinoplasty and Control groups, as well as between the preoperative and postoperative in the paper by Arima et al.^1^, enables us to imagine that the versions utilized may have responsiveness, which is one of the criteria required for validation.

## CONCLUSION

In the score from zero to 24 of the *Rhinoplasty Outcome Evaluation* in Brazilian Portuguese, we found 12 as the minimum normality limit, with sensitivity and specificity indices of 95.16% and 95%, respectively.
